# *Volvamos Juntos*: evaluation of the implementation of a Social Health Intervention to mitigate the impact of Covid-19 in businesses in Antofagasta, Chile

**DOI:** 10.1186/s12889-025-21297-3

**Published:** 2025-03-14

**Authors:** Jaime C. Sapag, Mónica Molina, Mayra Martínez, Paola Cordón, Patricio Céspedes, Mauro Concha, Marcelo Fuentes, Andrea Fernández, María Soledad Zuzulich, Paula Repetto, Guadalupe Echeverría, Hernán Cáceres, Blanca Peñaloza

**Affiliations:** 1 Department of Family Medicine, School of Medicine, Faculty of Medicine, Pontificia Universidad Católica de Chile, Santiago, Chile; 2 School of Public Health, Faculty of Medicine, Pontificia Universidad Católica de Chile, Santiago, Chile; 3 Dalla Lana School of Public Health, University of Toronto, Toronto, ON Canada; 4 Centre for Addiction and Mental Health, Toronto, ON Canada; 5 School of Psychology, Faculty of Social Sciences, Pontificia Universidad Católica de Chile, Santiago, Chile; 6 Department of Industrial Engineering, Faculty of Engineering and Geological Sciences, Universidad Católica del Norte, Antofagasta, Chile; 7 School of Nursing, Faculty of Medicine, Pontificia Universidad Católica de Chile, Santiago, Chile; 8 Center for Molecular Nutrition and Chronic Diseases, School of Medicine, Faculty of Medicine, Pontificia Universidad Católica de Chile, Santiago, Chile; 9 Department of Construction Management, Faculty of Engineering and Construction Sciences, Universidad Católica del Norte, Antofagasta, Chile; 10 School of Engineering Science, Industrial Engineering and Management (IEM), LUT University, Lappeenranta, Finland; 11 Department of Global Health and Population, Harvard T.H. Chan School of Public Health, Harvard University, Boston, United States of America

**Keywords:** Innovation, Evaluation, Implementation, COVID-19, Social health intervention, Businesses

## Abstract

**Background:**

The COVID-19 pandemic has had an impact not only on healthcare but also on labor and socioeconomic sectors worldwide, leading to the development of strategies to mitigate the crisis’ widespread repercussions. In Antofagasta, Chile, an innovation project entitled *Volvamos Juntos* (“*Let’s Return Together”*) was developed to support a diverse group of micro and small businesses. The project consisted of accompanying companies in the process of reopening safely and included interventions ranging from educating and testing employees for COVID-19 to developing protocols to avoid contagion and other preventive measures. The evaluation of the project’s implementation is presented here.

**Methods:**

A mixed-methods, collaborative study was conducted, adhering to the *Consolidated Framework for Implementation Research (CFIR)* and Proctor’s *Implementation Outcomes*, with an online survey, interviews, and focus groups with businesses’ representatives, the implementation team, and program stakeholders. Quantitative analyses were descriptive: frequencies and means were calculated, along with dispersion measures as appropriate, and in some cases, ANOVA tests were performed to assess differences. Qualitative information was processed with content analysis. Finally, an integrated hybrid analysis was conducted, guided by the study’s objectives and theoretical framework.

**Results:**

A total of 156 leaders from 203 participating businesses answered the online survey (response rate: 76.8%), and 46 people participated in the qualitative component (31 in interviews, 15 in focus groups). Overall, the **program’s implementation** according to different CFIR dimensions and certain outcomes was evaluated satisfactorily. In the survey, 96.7% participants rated the program’s **suitability** as satisfactory to maximum (grades 5 to 7 out of 7), 92.3% rated the **feasibility** with an average of 6.0, 97.4% rated the **sustainability** with an average of 5.9, and 94.3% indicated that they would favorably recommend (grades 6 or 7) the program to other institutions. Strengths and weaknesses were identified, and lessons learned include the need to plan for changing contexts, the relevance of collaborative and interdisciplinary work, and the importance of flexible support processes that promote autonomy and sustainability.

**Conclusions:**

*Volvamos Juntos* met its proposed implementation objectives, despite several challenges. Reflections from this innovative social health program are relevant for the development of other interventions in times of crisis.

**Trial registration:**

N/A.

## Background

The COVID-19 pandemic has had an unprecedented impact throughout the world, forcing national and international organizations to rapidly develop strategies to confront and mitigate the health crisis. A review of the literature reveals that authorities across countries took varied steps in their efforts to flatten the contagion curve [1].

In Chile, measures to control the transmission of COVID-19 from 2020 to 2021 were focused mainly on restricting the mobility of the general population, by suspending non-essential activities and enacting quarantines, which, in turn, had a progressively negative impact on the economic situation and on mental health [2].

In Latin American and the Caribbean, the implementation of these measures was challenging, given that it is “the most unequal region of the world, and also the most urbanized among developing regions, which exposes a significant portion of the population to contagion due to vulnerable conditions” [1].

The pandemic disrupted an already complex economic and political scenario in Chile, considering the social unrest and demands stemming out of the large-scale protests that shook the country in late 2019. The emerging health crisis beginning in March 2020 further exacerbated the negative impact on the population’s health and employment situation [3].

During the first year of the pandemic, the Chilean government established a series of initiatives to protect the population, such as strengthening the health system, creating tools to support families, restricting activities and mobility, and developing a testing, tracking, and isolation strategy, among others [4]. National health authorities implemented strict restrictions to reduce the risk of contagion in different parts of the country, including enforcing temporary quarantines that limited mobility and only allowed individuals to leave their homes for selected authorized, essential activities a couple times per week [4].

These restrictive measures compounded the pandemic’s negative impact on businesses across the board, which were already struggling due to the high rates of contagion. Not all businesses, however, were equally affected; microenterprises were the hardest hit, both in terms of the number of companies that experienced a drop in sales (63.1%) and in the magnitude of that drop (-37.5%) [5]. These effects also affected the workers themselves, who saw their wages decrease or, worse, were let go [5].

The impact of the pandemic also varied geographically, based on the characteristics of economic development in different regions of the country [6]. The Region of Antofagasta (Region II) in northern Chile is known for its mining activity: according to the 2009–2020 Antofagasta Regional Development Strategy, the mining sector accounts for more than 55% of the regional GDP [7]. There are, nevertheless, other sectors that make significant contributions, including construction, general services, transportation, communications, hospitality, and the restaurant industry. As much, micro and small businesses play a fundamental role in the regional economy.

**Fig. 1:**
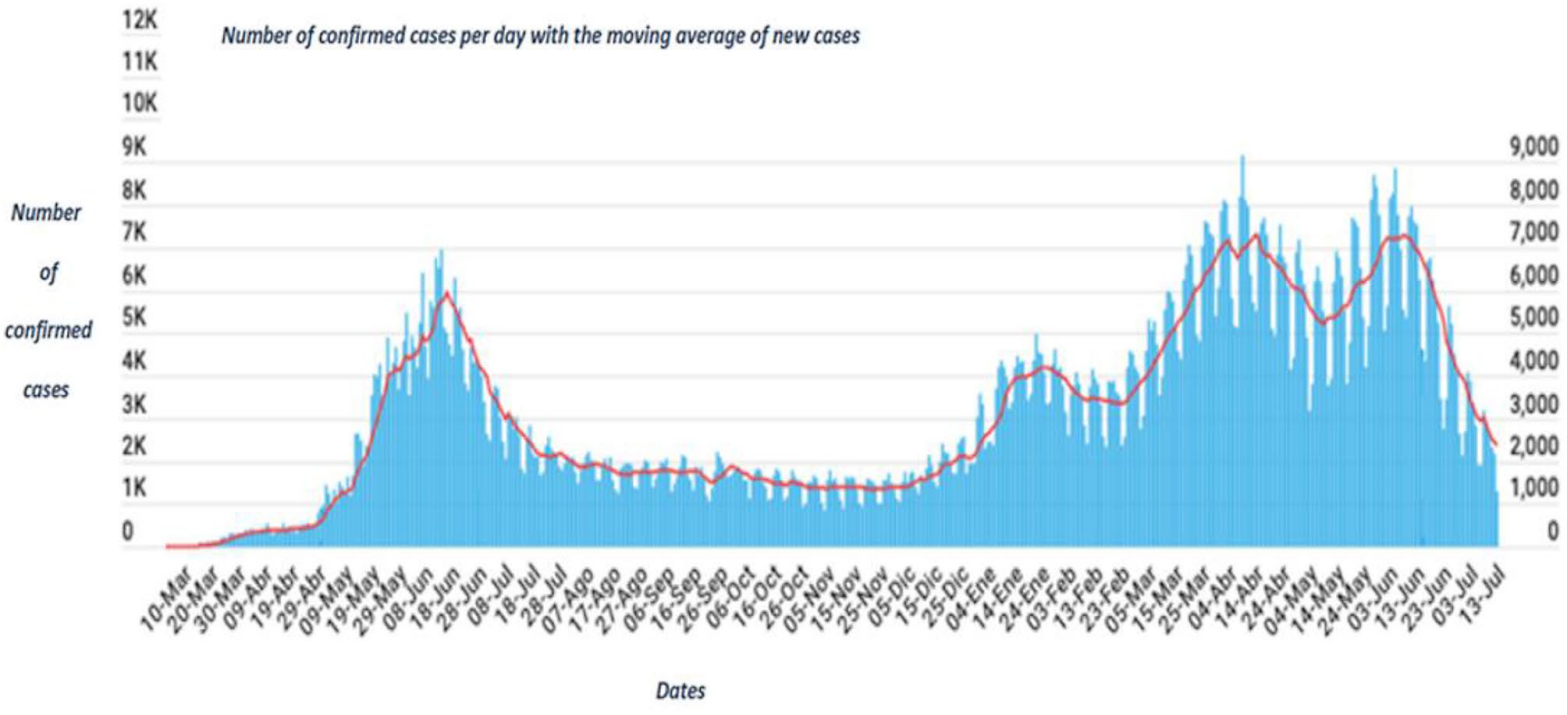
New cases of COVID-19 in the municipality of Antofagasta between March 10, 2020 and July 13, 2021

The capital of the Antofagasta Region, which shares its name, has a population of 361,873 inhabitants, according to the latest census [8]. The city of Antofagasta experienced multiple peaks of COVID-19 cases between 2020 and 2021, and the period between March and June 2021 had the highest incidence of cases (see Fig. 1).

**Fig. 2:**
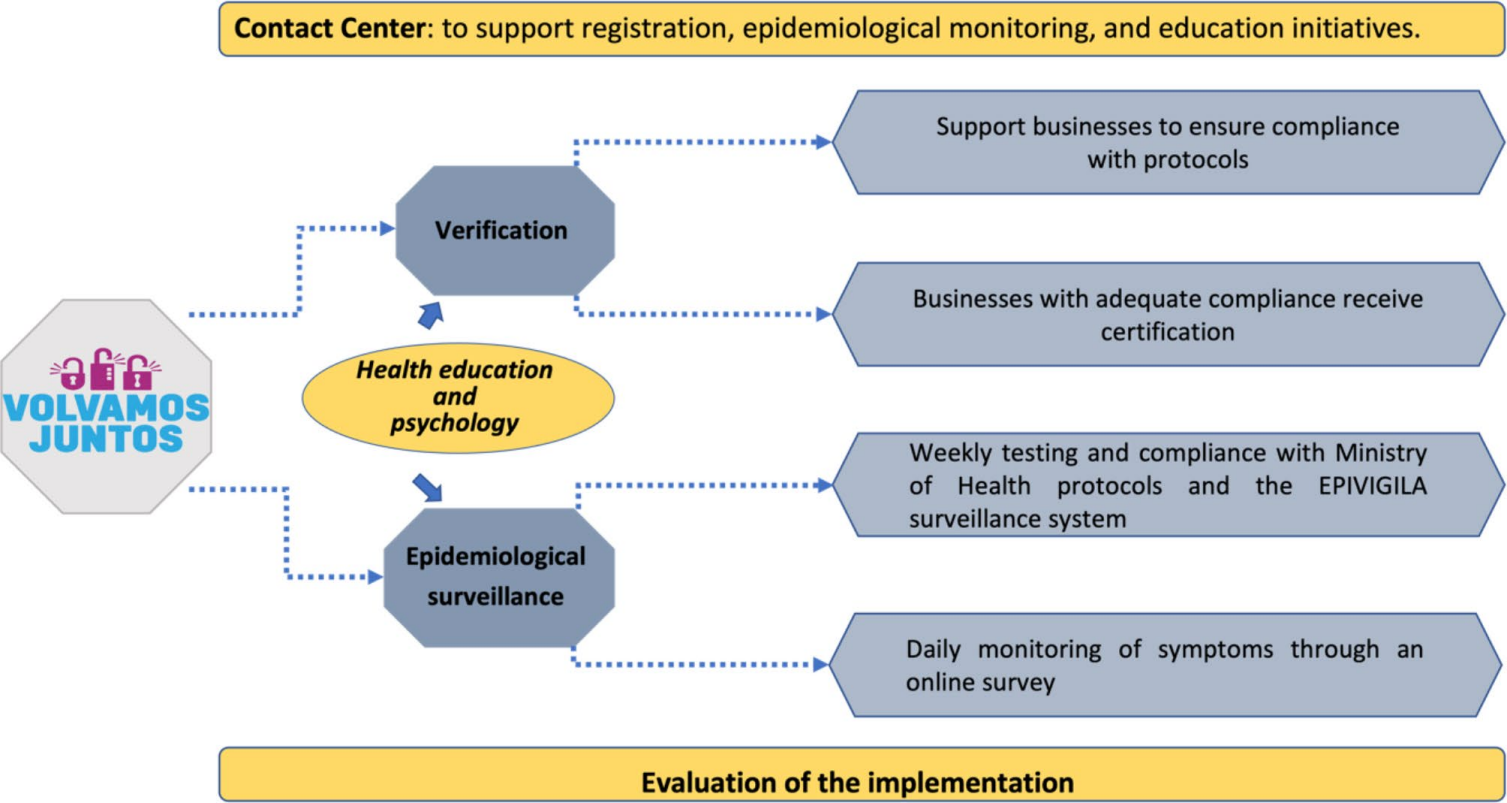
Components of *Volvamos Juntos program*

In this context, during the pandemic, three universities and BHP, a private mining company, joined together to spearhead an innovative and interdisciplinary program entitled *Volvamos Juntos* (“*Let’s Return Together”* in Spanish), whose general objective was to develop and implement a pilot program to prevent infections and facilitate the early detection of cases in order to support the safe reopening of micro and small businesses in the city of Antofagasta (details of the program in Table 1; Fig. 2).

**Table 1:** Characteristics of the program

**General objective**: To develop and implement a pilot initiative to prevent infections and facilitate the early detection of COVID-19 cases to support the safe reopening of micro and small businesses in the city of Antofagasta, in northern Chile (Antofagasta Region).
**Specific objectives**: 1) Develop and implement an external system to verify the correct implementation of Ministry of Health workplace protocols to prevent COVID-19 infections in participating businesses. 2) Implement an epidemiological surveillance system, using strategies of periodic testing in situ, screening for symptoms, and regularly evaluating high-risk contacts among employees in the participating businesses. 3) Develop and implement a plan to educate and support the participants, in order to increase adherence to the COVID-19 prevention measures.
**Location**: City of Antofagasta, Antofagasta Region.
**Intervention**: Multi-component program with interdisciplinary teams who implemented the processes of certification, epidemiological surveillance, community education, and evaluation of the implementation.
**Implementation**: Recruitment began in businesses that agreed to participate in the program through a registration and informed consent process. Businesses were visited periodically to prepare and implement a plan for safe work environments, and in parallel, workers were tested weekly. When positive cases were identified, workers were given clinical guidance on how manage the quarantine period and symptoms, with referral to health services if required. Additionally, online courses and informal talks were carried out in workplaces to educate businesses and workers about preventing infection and the correct use of personal protective equipment, as well as to provide information about the virus and its behavior.
**Evaluation**: Indicators were measured on a weekly basis, and the implementation of the program was evaluated using a collaborative mixed-methods study.

The program reached a significant population, with a total of 424 registered businesses, of which 203 were small and medium sized enterprises, and 65% were led by women. There were 5,210 direct participants (as of November 22, 2021), and approximately 20,840 people were impacted indirectly by the program (considering a family group of 4 people on average per participant) (as of November 22, 2021).

The main program achievements per component, based on indicators, are as follow: (1) 266 businesses were certified in correct compliance with ministerial COVID-19 protocols, (2) 18,886 COVID-19 tests were carried out (as of November 29, 2021), and (3) the education component trained a total of 788 people. Additionally, educational support materials were created to share on social media and had 9,538 views (as of July 29, 2021).

Considering the importance of the program scope, and its relevance for the community, the coordinating team decided to evaluate not only the achieved results but also the implementation process. For this reason, during the design process, an evaluation component was incorporated to identify the strengths and weaknesses of the program, as well as main lessons learned to permit the ongoing modification and improvement of *Volvamos Juntos* during the course of its implementation, to ensure maximum impact and reach.

The **objective** of this article is to present a synthesis of the results of the evaluation of the implementation of the *Volvamos Juntos* program, and identify the main lessons learned. The findings may be relevant for innovations in the context of future social health emergencies.

## Methods

**Fig. 3:**
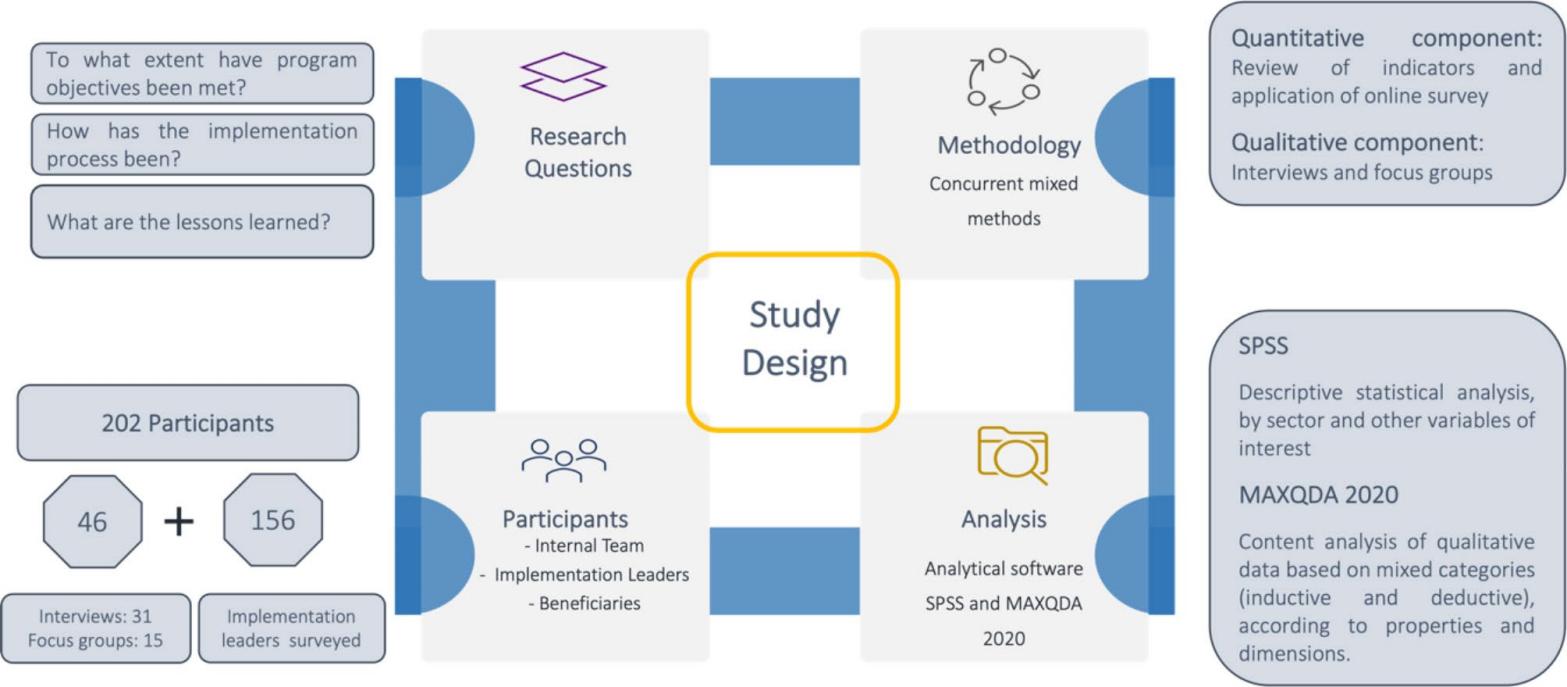
Synthesis of the design of the evaluation of the implementation of *Volvamos Juntos*

The **design** of the evaluation of the implementation of *Volvamos Juntos* was collaborative and used a mixed methods approach (see Fig. 3).

The evaluation component was guided by three specific objectives: (1) characterize compliance with the program’s objectives, (2) evaluate the results of the implementation of the intervention and associated products, in terms of their potential impact and contribution to the population of Antofagasta, and (3) identify lessons learned and recommendations.

Four **principles** guided the development of this evaluation: (1) Use of the results for the continuous improvement of the intervention, (2) Relevance to the local context, (3) Involvement of the community and stakeholders, and (4) Ethical and high-quality standards throughout the evaluation process.

The **theoretical foundation** that underlies the design, analysis, and presentation of the results is based on Implementation Research, which is the “application of scientific questioning to implementation, the act of putting an intention into practice, which in health research corresponds to the policies, programs, and practices (collectively called interventions)” [9]. *Implementation Science* contributes to understanding the what, the why, and the how of an intervention, in this case *Volvamos Juntos*, to determine how it works (or does not) in real contexts.

One of the most developed approaches in Implementation Science is the Consolidated Framework for Implementation Research (CFIR), which identifies five central areas that are relevant for implementation: characteristics of the intervention, internal context, external context, key involved individuals, and the implementation process [10]. Moreover, analysis of the *Implementation Outcomes* identified by Proctor et al. – acceptability, adoptability, suitability, feasibility, fidelity, implementation costs, coverage, and sustainability – indicate the degree of success of an implementation [11].

The study’s mixed methodology included preparing and conducting an online survey, as well as interviews and focus groups. The purpose of the **online survey** was to learn the opinions of the implementation leaders, who were owners or workers in the participating businesses that assumed the responsibility of promoting and implementing *Volvamos Juntos*. The survey inquired about the central aspects of the program in detail, including satisfaction with the implementation process, contributions to health care, flexibility, achievement of the objectives by component, and lessons learned. The implementation team piloted the survey before it was officially launched, to evaluate its general functioning.

An application plan, with three main steps, was designed to guide the application of the survey. The first step was dissemination of the survey through social networks (Facebook and Instagram), to present the program and survey to field teams and the target population, in order to emphasize the importance of the survey and their participation in the process. Secondly, the survey was distributed via e-mail and WhatsApp to businesses by the Contact Center, which also sent regular reminders, in accordance with the weekly follow-up plan. The third and final step was survey retrieval, which consisted of contacting participating businesses after 4 weeks, with the support of the field team, to insist they complete the online survey. The evaluation team also made personalized phone calls to the most resistant teams to increase survey coverage. This process was carried out between April 12 and July 6, 2021, and data was collected in a secure web platform to store and protect the information, in compliance with the study’s ethical guidelines.

The **interviews and focus groups**, on the other hand, were conducted in parallel between April 27 and July 19, 2021. Qualitative information was collected using an intentional sample, with selection criteria based on the different roles in the project and on ensuring adequate representation of women. Three groups of key actors were formed: implementation team, business leaders, and stakeholders. Relevant actors were identified and contacted by phone or e-mail to invite them to participate in the study and then coordinate a date and time to meet. Prior to each interview and focus group, the informed consent form, approved by the ethical committee, was sent via Google Forms, and later personally presented to each participant.

The interviews and focus groups were semi-structured; a few pre-determined questions, based on the initial evaluation objectives, guided the conversations around these central themes.

### Analysis

The **quantitative analysis** was descriptive. Frequencies and means were calculated, along with dispersion measures, as appropriate for each variable. Likewise, a differentiated analysis by sector was conducted, to determine differences in response profiles. In some cases, statistical tests were performed to assess the observed differences and calculate the corresponding p-values. Analysis of Variance (ANOVA) tests were applied to compare hypotheses and determine whether there were statistically significant differences between the means of two or more groups. A p-value of < 0.05 was considered significant.

For the **analysis of qualitative information**, the interviews and focus groups were recorded and transcribed. The audios and transcripts have been kept in the evaluation team’s archives, in accordance with the regulatory period suggested by the ethics committee. Codes were used in the transcripts to permit the anonymity of participants while allowing for the identification of their roles in the program, based on the established key actor groups.

**Hybrid content analysis** was carried out using the transcripts as units of analysis, which were classified and coded according to CFIR’s objectives and dimensions [10]. The analysis consisted of (1) coding the broad nodes (domains and dimensions); (2) identifying common, divergent, and emergent elements from the information; and (3) comparing and condensing the information, in line with the study’s guiding questions. To ensure the rigor of the results, and validity of the interpretations, the entire evaluation team participated in the analysis process, to permit triangulation.

## Results

The online survey was answered by 156 leaders of the participating businesses, from a total of 203 businesses (a response rate of 76.8%). There were 31 people who participated in the interviews, along with 15 in the focus groups, for a total of 46 participants in the study’s qualitative component (see Table 2).

**Table 2:** Participants by gender and data collection method

Group of actors	Participants (*N*)	Women (*N* and %)	Data collection method
Verification team	**8**	4 (50%)	Focus groups
Health team	**7**	4 (71%)	
Implementation team (Field team)	**2**	0 (0%)	Interviews
Implementation team (Management and administrative team)	**11**	7 (77%)	
Business leaders (Micro and small businesses)	**10**	7 (70%)	
Stakeholders (BHP mining, universities, and regional and national authorities)	**8**	4 (50%)	
**Qualitative component total**	**46**	**26 (56.5%)**	
**Quantitative component total**	**156**	**99 (53.46%)**	Online survey

The integrated results based on the three evaluation questions are presented in the following sections.

### Fulfillment of objectives and general satisfaction with the *Volvamos Juntos* program

Quantitively, the level of **fulfillment of the central activities** of the program was evaluated with an average score of 6.0 (on a scale of 1 to 7), which suggests that the businesses favorably assessed the different components. With regards to the **perceived contribution of the educational activities to the prevention of COVID-19**, the average score was 6.1.

Results of the qualitative analysis indicate that the participants had a positive assessment of *Volvamos Juntos*. The most highly valued aspect was the delivery of evidence-based knowledge about the virus and precautionary measures. The adoption of preventive strategies and workplace protocols were also highlighted, along with the team’s improved capacities.

*“Thanks to the testing*,* a young man who works with me was found to be positive … we work closely together*,* and given that close contact*,* we were all sent home. But imagine if we haven’t had your support*,* then we would have all been infected” (LE/Company Implementation Leader*,*11).*

In terms of **satisfaction** with “Volvamos Juntos”, 85.9% of the participants had a high impression of the program (grades of 6 or 7), with an average satisfaction of 6.1 (out of 7.0). Regarding the perception of the program’s contributions, 92.3% evaluated the program with a 6 or 7 for having supported the health of the workers in their businesses during the pandemic, with an average score of 5.9.

Furthermore, analysis of the qualitative data found that the participants valued: 1) the positive impact of the program’s activities on both the individual and community levels; 2) the program’s contributions to public policies during the particular social health context; and 3) the educational component, local capacity building, and management and development of these types of projects with the participating university and private sector collaborators.

*“I think that this initiative is very important*,* firstly because it is a public-private-academic collaboration*,* that has proven to be highly efficient and effective in meeting the needs and providing rapid response in this context*,* which is so changing*,* so uncertain*,* and so unknown” (SH/university decision-maker:7)*.

*“Since this is new*,* nobody knew about COVID. Some said one thing*,* others another*,* but they [the program] were teaching us about it. We took better care of ourselves with the distancing… and they supported us in enforcing the protocols” (LE/ Company Implementation Leader:27)*.

### Implementation of the program

In relation to the implementation of *Volvamos Juntos*, the results are presented according to the five central CFIR categories, including emergent areas that strengthened the process. (see Table 3)

1. *External context*: The study team was faced with challenges in both the program design and implementation processes, as they adapted to the difficult reality of the businesses, which were already in a weak position prior to the pandemic, due to the social conflicts that affected Chile at the end of 2019 and led to the partial closure of businesses, as well as looting and property damage in some cases. The businesses were then further affected by the restrictions and unpredictable, changing measures taken by the government in response to the COVID-19 pandemic.
2. *Internal context*: In light of the imposed quarantines, the initial recruitment strategy was modified to expand the profiles of companies eligible to participate in the study. The team’s coordination efforts and positive internal dynamics were essential strengths throughout this period and made it possible to maintain the study’s credibility and productivity with the local business and key actors.
3. *Characteristics of the intervention*: The program was a complex, multi-component intervention. The participants clearly distinguished between the functions of testing and certification and valued the contagion prevention measures, alongside the activities of the Contact Center and the health education component.
4. *Key individuals*: Different groups of key actors were identified.
  - Stakeholders. The managers and coordinators of the intervention, who were an interdisciplinary group that stood out for their leadership, flexibility, and high level of knowledge and skills in managing problems that arose; they work in academia and had access to up-to-date evidenced based information and institutional support.
  - Field team. Group of young professionals, most of them recent graduates, who used their personal and professional skillsets to respond to the needs of the project.
  - Implementation leaders of the businesses. Their leadership facilitated the adaptation and compliance to the intervention.
5. *Implementation process*: The evaluation process identified and characterized four strategic elements that strengthened the intervention, apart from the program components. These were: (1) flexibility to adapt the intervention to the local context, (2) training based on the needs of the different sectors, (3) a collaborative approach between the components, and (4) regular in-field support for the participating businesses.

**Table 3:** Selected quotes related the five categories

CFIR category	Excerpts from the transcripts
External context	“We ended the year with the social conflict, that hit us really bad. With the demonstration and protests, all the businesses closed, but then this other thing, the pandemic, happened, and we were really in trouble.” (LE*/Company Implementation Leader*:9, 12)
Internal context	“The issues with the quarantines couldn’t have been foreseen either, and this has led to a lot of uncertainty, and so inevitability we have had to make a lot of changes along the way.” (*ED/project leaders*:6, 25) “That is what I think has made the program successful, that you have handled everything in a humane and professional manner, very appropriately, and I think that we have also done well in that sense, with a lot of openness, with lots of management, which is important, so everything has worked out very well.” (*SH/Company decision-maker*:4, 39)
Characteristics of the intervention	“What I liked most was the certification and training. Because although one knows what is right and necessary, sometimes there are too many details, so learning about the preventive measures was super important.” (*LE/ Company Implementation Leader*:8, 37) “I highlight the education, the support. The testing was quite important… because I believe that… that stopped a lot of infections in the business. I also highlight the responsibility shown by the people who came, and those who talked to us over the phone.” (*LE/ Company Implementation Leader*:16, 50)
Key actors	“A strength of the program is that one can do interdisciplinary work …. That is, we have risk preventionists, health workers, educators, psychologists, evaluators like you, like there’s a multidisciplinary team with the same objective. All the viewpoints are necessary to see how each of these contributes to the final objectives, so we listen to all the opinions.” (*ET*/*Innovation Implementation manager*:2, 24) “Personally, this project is my first work experience. It helped me develop. And I also highlight the teamwork, since I’ve had to solve certain problems in the field, and I leaned on my colleagues, and they were always, wow! At least in this project that were super open. So I did feel support by a team. I think that was the first I learned: teamwork, empathy, assertiveness, experience.” (GFET3/ *education sector focus group*:10). “It’s incorporated… I come every day and see that the thermometer is working. I organize clients of the stores when there are waiting lines… I clarify, as the administration, that there are certain conditions in order to work… I think that the combination is good, that we are trained, that we comply with what the program taught it, and apart from that, I pay attention…” (*LE1*,* 42*)
Implementation process	“We are extremely satisfied… as it advanced, it stayed flexible…and nobody knows how the pandemic is going to evolve… it could adapt even though the quarantines were coming.” (SH5,13) “The health team [of the intervention] was also open to teach us, and answer our questions… about why the virus was mutating, why they first said this and then that. So they had a good disposition to respond…” (*LE/ Company Implementation Leader*:17,44). “The fact that three universities have come together to carry out a project that benefits the community and economy in Antofagasta, in association with a company like BHP, that they’re concerned about the issue and provide us resources… I think that this private-public link is very positive, that it’s not so usual, that all those involved share a commitment and contribution to the public. That’s very important.” (*SH/University decision-maker*:3,5)

### Implementation outcomes

The *implementation outcomes* served as indicators of the success of the implementation of *Volvamos Juntos*, and the qualitative analysis yielded information about these outcomes.

In terms of **acceptability and adoptability**, business leaders decided to enroll in the program due to the high recognition of the involved institutions, the fact that participation was free, and the benefits the program provided.

With regards to **suitability**, the characteristics of the intervention were perceived as appropriate. The participants highlighted the study’s in-field support and delivery of essential materials (posters, hand sanitizer dispensers, thermometers, etc.) that were not supplied by government institutions yet were mandatory to keep businesses open. They also shared that the periodic preventive testing allowed them to have peace of mind and keep their workplaces and homes safe from COVID-19.

*“I thought [the program] was quite good*,* because it was the support we needed… it’s the first time that we’ve been in a pandemic*,* and the program team was open and willing to teach and educate us about what was going on.” (LE/Company Implementation Leader:5*,*5).*

With respect to **fidelity**, results indicate that the program was carried out in compliance with the initial agreement signed by the participants and institutions. The educational component was especially underscored by the leaders and workers, for helping them achieve greater adherence to the recommendations outlines by the epidemiological surveillance and protocol certification teams.

*“They began to carry out weekly controls*,* with testing… and this continued every week*,* checking and filling out the forms they gave us*,* in addition to the survey that was messaged to our phones each day*,* in case you had any symptoms*,* to keep preventing contagion.” (LE/Company Implementation Leader:1*,* 6)*.

Regarding **feasibility and sustainability**, the businesses considered the program feasible to implement and voiced the need to maintain the measures implemented during the program. The different participating groups also believed that *Volvamos Juntos* would need to provide ongoing support and advice, given that the pandemic continues to have an important effect worldwide, meaning the businesses still need to stay up to date on that situation.

*“I think that what helped us was the correct use of the masks*,* the hand washing*,* which is super important*,* the social distancing without question. I think that the basic measures became engrained in each of the localities. I think that once you learn it well*,* it sticks with you.” (LE/Company Implementation Leader:1*,*36)*.

The quantitative results complement the participants’ perceptions about the *outcomes*. The **suitability** (flexibility) of the program to adapt to needs of each business and institution were rated as satisfactory to maximum (grades 5 to 7) by 96.7% of the participants. **Feasibility** (importance) was assessed similarly, with an average score of 6.0 by 92.3% of the participants, while **sustainability**, in terms of maintaining the changes achieved by the program over time, was evaluated with an average score of 5.9 by 97.4% of the responses. This is incredibly important, given that preventive measures against COVID-19 continue to be very relevant. With regards to the willingness to recommend the program to other intuitions, 94.3% of the respondents indicated favorable responses (grades of 6 and 7).

Emerging findings about the implementation of the program, focused on the prevention of COVID-19 with a population focus, highlighted several key aspects. These “legacies” of the program include the testing methods and risk management in the workplace; the work of Contact Center to provide supportive follow-up and advice to individuals with positive cases of COVID-19; designing and providing materials and resources to support compliance with protocols; training focused on the virus, transmission, and preventive behavior; and the collaborating institutions’ emphasis on local capacity building to develop the innovation program.

In line with the principle of continuous improvement, that supported the evaluation process, a number of **lessons learned** were identified, in the hope that they could support the successful implementation of similar initiatives in the future. These lessons include ensuring the quality of internal and external communication processes, designing unified tools to register each component of the program, and anticipating the challenges posed by implementing programs in diverse local contexts by planning for different scenarios.

Finally, the participants acknowledged the importance of developing a rigorous and collaborative **evaluation process** through the program’s implementation. This was highlighted by various study participants, as exemplified by the following quotes.

*“I love [the evaluation process]… that the evaluation team was involved in field from the very beginning. Because in the end*,* these experiences*,* to the extent that they are better recorded*,* evaluated*,* and objective*,* allow us to learn more and do better…and finally that everything we do is for the benefit of the population.” (SH/ university decision-maker:2*,* 34)*.

*“For me*,* it has been very innovative. I had worked in other projects*,* in other fields*,* and having this foundation of evaluation*,* to show both the duties and tasks of the internal team*,* as well as their activities*,* seems to me to be an element that I am always going to keep in mind in other projects … Introducing this central foundation of evaluation throughout the project*,* I feel that it contributed a lot*,* and assertively*,* to be able to adjust these processes… it allows us to not make mistakes*,* and adequately capitalize on the knowledge gained…” (ED/project leaders: 3*,* 38)*.

## Discussion

The evaluation of the *Volvamos Juntos* program, through a mixed methods approach, has led to a characterization of the implementation process and the identification of lessons learned, as well as opportunities for potential scale-ups in the future. It can be concluded that the program adequately achieved its established objectives. The initiative’s impact in helping businesses manage the care of their staff and families, thereby reducing the risk of contagion, and at the same time, facilitating the safe return to labor and educational activities, was positively assessed by the study participants. In addition, it may be possible that, because of all the implemented measures, clients were more willing and open to frequent the businesses, as they perceived greater concern for preventive measures.

Throughout Latin America, social protection programs have had to be developed and strengthened in light of the complex challenges posed by the pandemic [12]. *Volvamos Juntos* was implemented under very particular circumstances, with an elevated degree of uncertainty, which meant that the ability to be flexible and adapt the program to the dynamic realities of the participating businesses was essential. Other studies have confirmed the need for **flexibility** in programs that are implemented in times of crisis [13, 14]. With respect to the actors involved, the qualitative component of the evaluation identified three key roles associated with the initiative’s success: the stakeholders and experts, the *Volvamos Juntos* implementation team, and the local implementation leaders and teams. These individuals stood out for their collaborative efforts – supported by the leadership – in progressively consolidating a sustainable program, which required trust between the involved parties and commitment to the common objective. The **leadership profile** was another crucial variable for the success of the program’s implementation, as evidenced by published literature [15, 16]. It is suggested that strategic implementation leadership facilitates general implementation [15] and both individualistic and collective approaches to leadership are required in healthcare services [16].

The actors who participated in the evaluation of the program perceived a high level of **adherence** to the proposed activities, which is one of the key *outcomes* in the field of implementation science [17]. Some obstacles, however, were initially encountered – as suggested by the qualitative results of this evaluation - with the epidemiological surveillance component, especially with regard to COVID-19 testing and the daily symptom surveys, since it was difficult to obtain the expected response frequencies and maintain them over time to ensure greater impact. This could be due to time constraints and to businesses’ lack of experience incorporating preventive strategies during the workday. As the program progressed, strategies were developed to confront these challenges. It is also important to note that the high costs associated with the testing component makes its wide use difficult. This, together with the difficulties of adherence, should motivate the consideration of less expensive interventions, such as education.

Likewise, certain **weaknesses in recording and managing relevant data from the components** were identified, which made it difficult to better monitor progress and address critical aspects. As a result, for instance, the *Volvamos Juntos* project collected less data on its epidemiological surveillance component, as it was suggested before. It is common that programs that are rapidly developed during pandemics encounter this problem, which is why it is important to take measures to improve this process [18]. For instance, early in the pandemic COVID-19, as it was a novel medical condition without adequate guidance to support data capture, it took time for teams to incorporate some data into electronic health records and for providers to become familiar with new codes and to address information challenges [18].

Respondents to the evaluation component of *Volvamos Juntos* highlighted aspects they regarded as essential for the project’s successful implementation. **Perseverance** in identifying emerging needs and opportunities to continually improve the program, and the progressive integration of changes, facilitated the progress and consolidation of the initiative. In addition, focus on the **development of a bond** with the businesses, that was based on trust and mutual respect, contributed to the partnerships and reciprocal communication, as did the progressive delegation of responsibility to the businesses, which allowed innovations such as self-testing and ventilation improvements to be incorporated in a sustainable way. The quality of the relationships between actors participating in the implementation of complex innovations is absolutely essential [19]. Higher levels of program engagement tend to be related with better results [19], as it was also identified in this study.

Both the implementation team and the participating business that assumed key roles in the program went through **processes of progressive maturation and empowermen**t. Participants in the current evaluation regarded the commitment and contributions of the implementation team and participating businesses as key to meeting the project’s objectives. In Implementation Science, “maturation” is viewed as a key element for the consolidation and sustainability of innovations [20]. The maturity of practices in the health care domain directly enhances the quality and efficiency of health care services [20]. The findings of this implementation evaluation suggest that “maturation” has been a relevant ingredient for success.

Emerging findings about the implementation of the program, focused on the prevention of COVID-19 with a population focus, highlighted several key aspects. These “legacies” of the program include the testing methods and risk management in the workplace; the work of Contact Center to provide supportive follow-up and advice to individuals with positive cases of COVID-19; designing and providing materials and resources to support compliance with protocols; training focused on the virus, transmission, and preventive behavior; and the collaborating institutions’ emphasis on local capacity building to develop the innovation program.

Among the lessons learned, the **social purpose** that infused the project’s objectives and principles was highly valued, along with the **interdisciplinary work** that brought together health professionals, experts in risk prevention, managers and administrators, and psychologists to serve the multiple needs of the participating workplaces. At the same time, the businesses and institutions see *Volvamos Juntos* as a real ally, which helped them have the security they needed to face the challenges of the pandemic. For their part, stakeholders underscored the importance of **collaborative work**, and how this can contribute to enhancing the social values of institutions and magnify their actions with a much greater impact, than if they were done individually. Collaborating in the implementation of programs is a key element to achieve success, and there are various ways to pave the way for intersectoral partnerships [21]. Then, developing specific collaboration strategies from the beginning may be critical for enhancing the results when implementing cross-system interventions, such as *Volvamos Juntos*.

With respect to the **strengths** of the study, it is pertinent to recognize the role of having developed a rigorous, collaborative evaluation process throughout the entire process of implementing the *Volvamos Juntos* program. The wide diversity of participants in the evaluation process, the evaluation team’s ability to spend time “in the field” in Antofagasta, the variety of data collection methods, the rigor of the evaluation process, and the triangulation of data were assets of the program. Having had well-defined principles to guide the evaluation, along with a well-defined theoretical-conceptual framework that strongly considered the field of Implementation Science, also contributed to the adequate and pertinent development of this evaluation.

Among the **limitations** of the study, it is important to include the challenges posed in evaluating a rapidly evolving initiative, in the midst of a pandemic, as well as the fact that it was not possible – and potentially unethical – to have businesses participate as a control group, and thereby not implement the program. It is also necessary to indicate that the incorporation of the evaluation team in a continuous and close manner with the implementation group posed a risk of bias. Nevertheless, special precautions were taken to avoid and mitigate this risk as much as possible, including ensuring necessary autonomy of the evaluation team, as well as following ethical standards in the field during the evaluation process. Undoubtedly, the balance between “closeness” and “distance” in this process is always a challenge [22]. A Developmental Evaluation approach considers a closer connection between implementation and evaluation [23].

**In the future**, other areas in which collaboration between the institutions participating in *Volvamos Juntos* can contribute to the construction of solutions for other important problems derived from the pandemic, or beyond, should be considered.

In summary, this article confirms the successful implementation of a social health intervention during the COVID-19 pandemic to support the safe re-opening of businesses in Antofagasta, Chile, and it identifies the main lessons learned in the process. Many of these lessons could be applied to develop new programs locally in Chile, as well as in other locations across the Latin American region and around world, to adequately address the challenges that affect people in diverse contexts, including the workplace.

## Conclusions

*Volvamos Juntos* was adequately implemented and met its proposed objectives. This innovative social health program leaves relevant lessons for the development of other interventions during crises. Among them, the following lessons stand out: (1) the importance of including the perception of participants in the decision-making process, from the beginning of the program design; (2) designing unified tools that guide the implementation of the different components of the program; (3) anticipating the possibility of changing scenarios in advance, to plan with flexibility and dynamism; (4) ensuring the quality of internal and external communication processes; (5) creating support systems for local implementers; (6) implementing education processes that promote the autonomy and sustainability of individual and organizational behavioral changes; and (7) strengthening community-based, interdisciplinary work, as the foundation for the successful implementation of innovations.

## Data Availability

The datasets used and/or analysed during the current study available from one of the project leaders, Blanca Peñaloza (bpenalo@uc.cl), on reasonable request. There maybe some restrictions related with the directions of the Scientific Ethical Committee.
